# In vitro functional rescue by ivacaftor of an ABCB11 variant involved in PFIC2 and intrahepatic cholestasis of pregnancy

**DOI:** 10.1186/s13023-021-02125-4

**Published:** 2021-11-18

**Authors:** Elodie Mareux, Martine Lapalus, Amel Ben-Saad, Isabelle Callebaut, Thomas Falguières, Emmanuel Gonzales, Emmanuel Jacquemin

**Affiliations:** 1 Inserm, Physiopathogénèse et traitement des maladies du foie, UMR_S 1193, Université Paris-Saclay, Hepatinov, 91400 Orsay, France; 2 Muséum National d’Histoire Naturelle, UMR CNRS 7590, Institut de Minéralogie, de Physique des Matériaux et de Cosmochimie, IMPMC, Sorbonne Université, 75005 Paris, France; 3 Paediatric Hepatology and Paediatric Liver Transplant Department, National Reference Center for Rare Paediatric Liver Diseases, FILFOIE, ERN RARE LIVER, Assistance Publique-Hôpitaux de Paris, Faculté de Médecine Paris-Saclay, CHU Bicêtre, 94270 Le Kremlin-Bicêtre, France

**Keywords:** BSEP, Cholestasis, VX-770, Potentiator, ABC transporters superfamily, Paediatrics

## Abstract

**Background:**

*ABCB11* variations are responsible for a spectrum of rare liver diseases, including progressive familial intrahepatic cholestasis type 2 (PFIC2) and intrahepatic cholestasis of pregnancy (ICP). Current medical treatment of these conditions mostly relies on ursodeoxycholic acid with limited efficacy. We report on the in vitro study of the p.A257V missense variant of ABCB11 identified in a PFIC2 patient and in her mother who experienced ICP.

**Results:**

The Ala257 residue is located outside the ATP-binding site of ABCB11. We show that the p.A257V variant of ABCB11 is correctly expressed at the canalicular membrane of HepG2 cells but that its function significantly decreased when studied in MDCK cells. This functional defect can be fully rescued by Ivacaftor.

**Conclusion:**

Ivacaftor could be considered as a new pharmacological tool able to respond to an unmet medical need for patients with ICP and PFIC2 due to *ABCB11* variations affecting ABCB11 function, even when the residue involved is not located in an ATP-binding site of ABCB11.

**Supplementary Information:**

The online version contains supplementary material available at 10.1186/s13023-021-02125-4.

**To the editor:**

Variations in ATP-binding cassette subfamily B member 11 gene (*ABCB11*), also known as bile salt export pump (BSEP), encompass a spectrum of diseases ranging from progressive familial intrahepatic cholestasis type 2 (PFIC2) to intrahepatic cholestasis of pregnancy (ICP). Recently, we have shown that ivacaftor (VX-770), an ABC transporter potentiator with a marketing authorization in cystic fibrosis (Kalydeco®), was able to rescue the functional defect due to the Abcb11-T463I variant located in an ATP-binding site and identified in a PFIC2 patient [1]. In line with these results, we investigated here the Abcb11-A257V variant. We have previously reported that a compound heterozygous PFIC2 girl, harbouring this p.A257V variation of *ABCB11* on one allele, had a mother, heterozygous for this *ABCB11* variation, who experienced ICP [2]. Here, we report that the p.A257V variation may lead to an impaired function of the transporter, thus supporting the role of this *ABCB11* variation in the pathogenesis of ICP and PFIC2. The Ala257 residue is located outside ATP-binding sites, in the fourth transmembrane (TM) helix of ABCB11, at the level of the lateral opening of the translocation cavity to the membrane inner leaflet observed in the inward-facing conformation of the transporter (Fig. 1A). The p.A257V variation of *ABCB11* is predicted as “possibly damaging/deleterious” by Polyphen2 [3], SNAP2 [4] and PROVEAN [5] and as “disease-causing” by MutationTaster [6].

The Ala > Val variation does not drastically change the polarity and steric hindrance of this region of the transporter. However, it is located in a region that has been observed in human PgP/ABCB1 to undergo a local conformational change, from a flexible disordered state in the inward-facing conformation to a continuous helix in the outward-facing one [7]. Interestingly, Ala > Val variation in a similar TM4 position within *Cyanidioschyzon merolae* ABCB1 has been shown to diminish transport activity [8]. Moreover, when we superimpose the 3D structures of the two halves of ABCB11, we observe that Ala257 in TM4 stands at analogous positions than Thr919 in TM10 (Additional file 1: Fig. S1), an amino acid for which mutation has been associated with benign recurrent intrahepatic cholestasis type 2 [9]. In addition, this region of TM4/TM10 is flexible between the two transporter halves (Additional file 1: Fig. S1). This suggests that both residues may play a critical role in the ABCB11 local conformational changes between the inward- and outward-facing states and may thus indirectly impact its function. Indeed, although Abcb11-A257V variant appears as a mature protein (Fig. 1B; quantification in Fig. 1C) correctly targeted to the canalicular/apical membrane when expressed in HepG2 cells and MDCK cells (Fig. 1D-E), it displays a transport activity defect (Fig. 1F). In MDCK cells co-expressing Abcb11 at the apical membrane and sodium-taurocholate co-transporting polypeptide (Ntcp) at the basolateral membrane, we observed that the p.A257V variant retains only 68% of wild type taurocholate transport activity (Fig. 1F). This decreased activity due to the p.A257V variation may therefore explain a PFIC2 phenotype when associated with another deleterious *ABCB11* variation [2]. In a heterozygous state, this residual activity may be sufficient to ensure a satisfying level of biliary bile acid secretion in physiological conditions, excluding pregnancy. However, during pregnancy, the high level of circulating female sex hormones and metabolites can modify ABCB11 expression by repressing *ABCB11* transcription and decreasing normal allele expression [10]. This could have favoured the transient decompensation of the p.A257V heterozygous state leading to ICP [11].

**Fig. 1:**
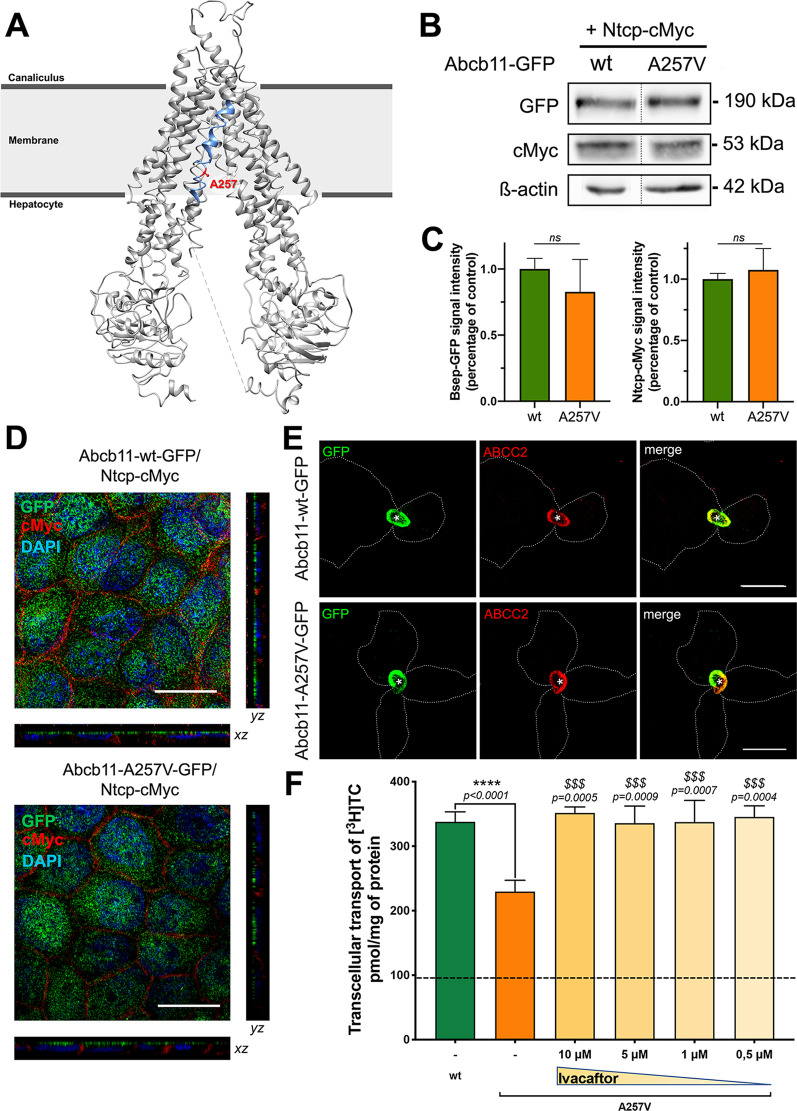
Characterisation of the p.A257V variant of ABCB11 and effect of ivacaftor on taurocholate (TC) transport activity. **A** Structure of wild type (wt) human ABCB11 in an inward-open state solved at 3.5 Å using cryogenic electron microscopy (PDB ID: 6LR0 [18]). The fourth transmembrane helix (TM4) is colored in blue and the Ala257 residue is depicted in red. **B** MDCK cells stably expressing Abcb11-GFP (wt or A257V) and Ntcp-cMyc were lysed and analysed by immunoblotting. This panel is representative of at least six independent experiments. **C** Electrophoretic patterns from B were separately quantified and their relative amounts were calculated and normalized to β-actin. Results are means (± SEM) of at least three experiments. *ns*: not significant (Student *t* test). **D** Abcb11-GFP (wt or A257V) and Ntcp-cMyc were stably expressed in MDCK cells. After immunolabelling, Abcb11 (green) and Ntcp (red) were visualized by confocal microscopy. Bottom, centre and right panels show *x–z*, *x–y* and *y–z* plane images, respectively. Bars: 10 μm. **E** Abcb11-wt-GFP and Abcb11-A257V-GFP were transiently expressed in HepG2 cells. After immunolabelling, Abcb11 (green) and ABCC2 (red) were visualised by confocal microscopy. *Bile canaliculus. Bars: 10 μm. **F** Vectorial transport of [^3^H]TC in MDCK cells stably expressing Abcb11 (wt or A257V) and Ntcp in the absence (−) or presence of the indicated concentrations of ivacaftor. The dashline indicates [^3^H]TC transport measured in MDCK cells expressing Ntcp alone. Means (± SEM) of at least six independent experiments for each tested condition are shown. ****P < .0001 versus wt; and ^$$^*P* < .01; ^$$$^*P* < .005 versus non-treated A257V cells (one-way ANOVA)

So far, the efficiency of ivacaftor on ABCB11 variants involving residues localized outside ATP-binding sites has not been demonstrated. However, since ivacaftor is approved in selected cystic fibrosis patients harbouring cystic fibrosis transmembrane conductance regulator variations localized inside and outside ATP-binding sites [12], similar effects might be considered for such types of *ABCB11* variations. Indeed, we report here that ivacaftor, even at low concentration (0.5 µmol/L), rescues the function of the Abcb11-A257V variant, increasing its transport activity, back to the one of Abcb11 wild type (Fig. 1F). These results are in line with previous reports showing that ivacaftor potentiation mechanism could also be independent of both ATP-binding and nucleotide binding domain dimerization [13]. It is also important to note that ivacaftor does not significantly modify the function of wild type Abcb11, as we recently reported [1]. Moreover, ivacaftor has been shown to rescue the channel activity of wt and mutated ABCC7/CFTR at subnanomolar concentrations in patch-clamp experiments [14], suggesting that it might be used at low doses in patients.

Based on these in vitro studies, including the present one, ivacaftor might thus be expected to result in clinical benefit in ICP and PFIC2 patients carrying missense *ABCB11* variations, such as p.T463I and p.A257V, on at least one allele. In addition, it has been reported that at least 5% of women experiencing ICP may harbour a heterozygous *ABCB11* variation [15]. Due to limited data during pregnancy, it is currently recommended to avoid or discontinue ivacaftor in pregnant women [16]. However, uncomplicated pregnancies have been reported in women with cystic fibrosis and treated with ivacaftor, leading to the birth of healthy neonates [16]. These reports suggest that ivacaftor could be used safely in pregnant women experiencing ICP. However, further studies are needed to support the use of ivacaftor in pregnant women. So far, ursodeoxycholic acid is the most commonly used and admitted treatment for ICP and remains the first-line therapy even if it is a matter of debate [17]. The present in vitro data constitute a proof of concept that ivacaftor has therapeutic potential for selected patients with PFIC2 and ICP caused by *ABCB11* missense variations affecting the function of the transporter. Considering that there is an unmet medical need for patients with BSEP deficiency, this could represent a significant step forward for the care of such selected patients.

## Supplementary Information

**Additional file 1.** Supplementary Materials and Methods and Figure S1 "Superimposition of the two transporter halves of the 3D structure of ABCB11".

## Data Availability

The datasets used and/or analysed during the current study are available from the corresponding author on reasonable request.

## Abbreviations

ABCB11: ATP-binding cassette subfamily B member 11
ICP: Intrahepatic cholestasis of pregnancy
PFIC2: Progressive familial intrahepatic cholestasis type 2
