# Supplementary material for: In vitro functional rescue by ivacaftor of an ABCB11 variant involved in PFIC2 and intrahepatic cholestasis of pregnancy
Source: Orphanet J Rare Dis. 2021 Nov 18;16:484. doi: 10.1186/s13023-021-02125-4 (PMC8600881; doi:10.1186/s13023-021-02125-4)
Supplement: Supplementary file 1 — Additional file 1. Supplementary Materials and Methods and Figure S1 "Superimposition of the two transporter halves of the 3D structure of ABCB11". [file 13023_2021_2125_MOESM1_ESM.docx]

Letter to the editor

***In vitro* functional rescue by ivacaftor of an ABCB11 variant involved in PFIC2 and intrahepatic cholestasis of pregnancy**

Elodie Mareux^1^, Martine Lapalus^1^, Amel Ben-Saad^1^, Isabelle Callebaut^2^, Thomas Falguières^1^, Emmanuel Gonzales^1,3^, Emmanuel Jacquemin^1,3,*^

^1^ Inserm, Physiopathogénèse et traitement des maladies du foie, UMR_S 1193, Université Paris‑Saclay, Hepatinov, 91400 Orsay, France.

^2^ Sorbonne Université, Muséum National d’Histoire Naturelle, UMR CNRS 7590, Institut de Minéralogie, de Physique des Matériaux et de Cosmochimie, IMPMC, 75005 Paris, France.

^3^ Paediatric Hepatology and Paediatric Liver Transplant Department, National Reference Center for Rare Paediatric Liver Diseases, FILFOIE, ERN RARE LIVER, Assistance Publique-Hôpitaux de Paris, Faculté de Médecine Paris-Saclay, CHU Bicêtre, 94270 Le Kremlin-Bicêtre, France.

^*^**Corresponding author**: Professeur Emmanuel Jacquemin, MD, PhD – Service d’Hépatologie et de transplantation hépatique pédiatriques – Hôpital Bicêtre – 78, rue du Général Leclerc – Le Kremlin Bicêtre – 94275 cedex, France. Phone: +33‑(0)1‑45‑21‑31-68; Fax: +33‑(0)1‑45‑21-28-16; e-mail: [emmanuel.jacquemin@aphp.fr](mailto:emmanuel.jacquemin@aphp.fr)

**Table of contents**

**Supplementary Materials and Methods 2**

*Patient, DNA construct and mutagenesis* 2

*Cell culture, transfection, lentiviral infection and immunoanalysis* 2

*Taurocholate transport assay and ivacaftor treatment* 2

*Statistical analyses* 3

**Supplementary Figure S1 4**

**Supplementary References 5**

**Supplementary Materials and Methods**

## Patient, DNA construct and mutagenesis

General information regarding the patients and *ABCB11* gene analysis have been previously reported.^1,2^ The C-terminus green fluorescent protein (GFP) plasmids encoding wild type (wt) and A257V missense variant of Abcb11 have been described, as well as the C-terminus cMyc plasmid encoding Ntcp.^2,3,4^

## Cell culture, transfection, lentiviral infection and immunoanalysis

Abcb11-encoding vectors (wt and A257V) were transiently transfected in HepG2 cells as published.^4^ Madin-Darby canine kidney (MDCK) cells were transfected, then stable transfected clones were sorted as described.^3,4^ MDCK clones with the highest Abcb11-GFP (wt or A257V) expression and parental MDCK cells were infected with lentiviral particles containing the Ntcp recombined plasmid as described.^3,4^ Immunofluorescence and immunoblotting analyses were performed as described,^3^ using the following primary antibodies: rat monoclonal anti-cMyc (Clone JAC6; GeneTex, Irvine, CA), rabbit polyclonal anti-GFP (ab290; Abcam, Cambridge, UK), mouse monoclonal anti-ABCC2 (clone M2I-4; Enzo life Sciences, Villeurbane, France), mouse monoclonal anti-GFP (clone 7.1 and 13.1; Roche Diagnostics, Manhnheim, DE), anti-cMyc (Clone 9E10; BD Biosciences Pharmingen, San Diego, CA) and anti-β-actin (clone AC-15; Sigma Aldrich, Saint-Louis, MO). Peroxydase- and fluorochrome-conjugated secondary antibodies were from GE Healthcare (Chicago, IL) and Molecular Probes/ThermoFisher Scientific (Illkirch, France), respectively.

## Taurocholate transport assay and ivacaftor treatment

Measurement of Abcb11-mediated taurocholate (TC) secretion was performed as described.^3^ In brief, culture medium was replaced by pre-warmed transport buffer in apical and basal compartments, in the absence or presence of 0.5 to 10 µmol/L ivacaftor (S1144, Selleck Chemicals, Munich, DE). [^3^H]TC was added in the basal compartment. After two hours, the apical buffer was collected and transcellular transport of [^3^H]TC was calculated from the radioactivity present in the apical buffer. Transport data were normalized to protein amounts determined by BiCinchoninic acid Assay (QuantiPro^™^ BCA Assay Kit, Sigma Aldrich, Saint-Louis, MO).

## Statistical analyses

Data are expressed as means ± standard error of the mean (SEM). Statistical analyses were performed using Student *t* test and one-way ANOVA. A *P* value *<* .05 was considered to be significant.

**Figure S1: Superimposition of the two transporter halves of the 3D structure of ABCB11.** The two transporter halves of the 3D structure of ABCB11 (pdb entry: 6LR0) were split and superimposed using UCSF Chimera.^5^ The two transporter halves are indicated in different colors, as well as the residues Ala257 and Thr919 in TM4 and TM10, respectively.

# Supplementary References

[1] Davit-Spraul A, Fabre M, Branchereau S, Baussan C, Gonzales E, Stieger B, *et al.* ATP8B1 and ABCB11 analysis in 62 children with normal gamma-glutamyl transferase progressive familial intrahepatic cholestasis (PFIC): phenotypic differences between PFIC1 and PFIC2 and natural history. Hepatology 2010;51:1645-5165.

[2] Gonzales E, Grosse B, Schuller B, Davit-Spraul A, Conti F, Guettier C, *et al****.*** Targeted pharmacotherapy in progressive familial intrahepatic cholestasis type 2: Evidence for improvement of cholestasis with 4-phenylbutyrate. Hepatology. 2015;62:558-566.

[3] Mareux E, Lapalus M, Amzal R, Almes M, Ait-Slimane T, Delaunay JL*, et al.* Functional rescue of an ABCB11 mutant by ivacaftor: A new targeted pharmacotherapy approach in bile salt export pump deficiency. Liver Int 2020;40:1917-1925.

[4] Amzal R, Thébaut A, Lapalus M, Almes M, Grosse B, Mareux E, *et al.* Pharmacological premature termination codon readthrough of ABCB11 in bile salt export pump deficiency: an in vitro study. Hepatology 2020;73:1449-1463.

[5] Pettersen EF, Goddard TD, Huang CC, Couch GS, Greenblatt DM, Meng EC, Ferrin TE. UCSF Chimera--a visualization system for exploratory research and analysis. J Comput Chem 2004;25:1605-1612.
